# Long-Term Assessment of AAV-Mediated Zinc Finger Nuclease Expression in the Mouse Brain

**DOI:** 10.3389/fnmol.2017.00142

**Published:** 2017-05-23

**Authors:** Muzna Zahur, Johan Tolö, Mathias Bähr, Sebastian Kügler

**Affiliations:** ^1^Department of Neurology, University Medical Center GöttingenGöttingen, Germany; ^2^Center for Nanoscale Microscopy and Molecular Physiology of the Brain at Department of Neurology, University Medical Center GöttingenGöttingen, Germany

**Keywords:** genome editing, zinc finger nuclease, cathepsin D, adeno-associated virus, *in vivo*

## Abstract

Gene editing tools like TALENs, ZFNs and Crispr/Cas now offer unprecedented opportunities for targeted genetic manipulations in virtually all species. Most of the recent research in this area has concentrated on manipulation of the genome in isolated cells, which then give rise to transgenic animals or modified stem cell lines. Much less is known about applicability of genetic scissors in terminally differentiated, non-dividing cells like neurons of the adult brain. We addressed this question by expression of a pair of ZFNs targeting the murine cathepsin D gene in CNS neurons by means of an optimized AAV viral vector. We show that ZFN expression resulted in substantial depletion of cathepsin D from neuronal lysosomes, demonstrating a robust gene deletion. Importantly, long-term ZFN expression in CNS neurons did not impair essential neuronal functionality and did not cause inflammation or neurodegeneration, suggesting that potent genetic scissors can be expressed safely in the mouse brain. This finding opens up new venues to create novel research models for neurodegenerative disorders.

## Introduction

Modern gene editing technology based on transcription activator-like effector nucleases (TALENs), clustered regularly interspaced short palindromic repeats-associated Cas9 (CRISPR/Cas) and zinc finger nucleases (ZFNs) has revolutionized opportunities for ablating or replacing genes in virtually every kind of organism (Ul Ain et al., 2015; Sovova et al., 2016). While some gene replacement studies have been conducted already on the organismic level, i.e., in the liver (Jo et al., 2015; Sharma et al., 2015), the vast majority of gene knockout strategies have been executed in cell-based systems, i.e., oocytes, cell lines or stem cells (Hermann et al., 2014; Rumi et al., 2014; Sung et al., 2014). Exploiting gene-modified founder cells not only offers the advantage of easy introduction of gene-editing tools, but also allows for their dilution during subsequent cell divisions, thereby limiting potential toxic or off-target effects of the genetic scissors. In contrast, very limited knowledge is available regarding the use of gene editing tools in non-dividing cells like neurons, which will accumulate the genetic scissors without possibility of diluting them by cell divisions. It is thus questionable if, e.g., a pair of ZFNs can be expressed in CNS neurons over long-term without inducing toxicity or immunological consequences. It needs to be taken into account that all TALENs, Crispr/Cas, and ZFNs largely consist of non-mammalian components, i.e., the DNA cutting bacterial enzyme moiety. It was reported for some Crispr/Cas systems that they cleaved DNA at a multitude of off-target sites (O’Geen et al., 2015) and that inappropriate expression in liver caused toxic effects (Guan et al., 2016).

A very promising genome modification approach is based on ZFN technology (Urnov et al., 2010). ZFNs are designer nucleases that consist of two functional domains: a customized zinc finger array that specifies DNA binding and the endonuclease domain of the restriction enzyme FokI that contains the catalytic activity. The zinc finger array in a ZFN subunit confers binding to the respective target half-site, and upon dimerization of the two subunits in correct spacing and orientation, the nuclease domain introduces a DNA double strand break (DSB) within the spacer sequence that separates the two target half-sites (Carroll, 2011). These DNA strand breaks are then repaired by the cell in a process called non-homologous end joining, which produces small insertions or deletions (indels), leading to a non-functional protein product. Design of ZFNs targeting exons in the very 5′-region of a mRNA prevent generation of larger non-functional protein moieties. CRISPR/Cas and TALENs are more attractive for many research applications in terms of the ease of design, cost effectivity and higher specificity. However, for more challenging tasks, e.g., human therapy, the development of the cleavage reagent represents a small part of the cost and effort devoted to the project. Considerations like specificity and ease of delivery then become paramount. In this regard, the smaller size of the ZFNs will offer an advantage in some circumstances. ZFNs targeted to the human CCR5 gene have been in clinical trials for several years and are proving safe and effective. Additional ZFN pairs have been targeted to other human disease, e.g., hepatitis B virus (HBV) gene (Weber et al., 2014) and hemophilia (Anguela et al., 2013).

The ability to express functional gene editing tools in the brain of experimental animals offers great promise to design research models impossible or very difficult to achieve by conventional knock-out strategies, i.e., if the gene deletion is embryonically lethal, has detrimental effects outside the central nervous system (CNS), or shall be generated only in a specific sub-population of brain cells. In the present study we used an AAV vector to transport a pair of ZFNs into mouse neurons *in vitro* and *in vivo*, and investigated long-term safety of this approach. The ZFN pair was designed to cleave the murine cathepsin D (CatD) gene. CatD is the cell’s major ubiquitous lysosomal protease (Benes et al., 2008), and impaired protein degradation is intimately linked to neuronal disorders like Alzheimer’s and Parkinson’s disease (Cullen et al., 2009; Tian et al., 2014; Vidoni et al., 2016). The potential contribution of CatD to neurodegenerative disorders could not yet be studied in detail, however, as conventional CatD KO animals die at very early age due to severe visceral phenotypes, especially in the immune system (Saftig et al., 1995). We thus aimed for a localized knockout of the gene in a relatively small subpopulation of striatal neurons. This approach is challenging insofar as CatD is partially secreted and can be uptaken by remote cells (Shevtsova et al., 2010), resulting in a supply source that might compensate the cells with AAV-ZFN system mediated CatD gene deletion.

AAV vectors are currently the preferred gene transfer and gene therapy tools due to their proven safety and efficacy record and availability of scalable production processes for clinical applications (Kotterman and Schaffer, 2014). Despite their limited transgene capacity, a pair of ZFNs can be fit into their genome, which is not possible for TALENs and difficult with current Crispr/Cas systems (Ul Ain et al., 2015). In addition to addressing safety of neuronal ZFN expression we thus aimed at designing an optimized vector genome layout, which allowed to package the ZFN pair into a single vector, thereby enhancing gene cleavage efficacy.

Our results show that the optimized single vector-expressed ZFN pair robustly diminished CatD expression in neurons without affecting essential neuronal functionality, and that after several months of expression in the adult brain no neurodegeneration or inflammation was observed, suggesting safety of long-term ZFN expression in the mouse CNS. This technology might become useful for ablating dominantly acting disease-related alleles, i.e., in Huntington’s disease or other CAG-repeat disorders, Rett syndrome, genetic cases of PD (LRKK2, SNCA) or AD (APP, PSEN1, PSEN2).

## Materials and Methods

### Plasmid Construction and Viral Vector Production

**FIGURE 1:**
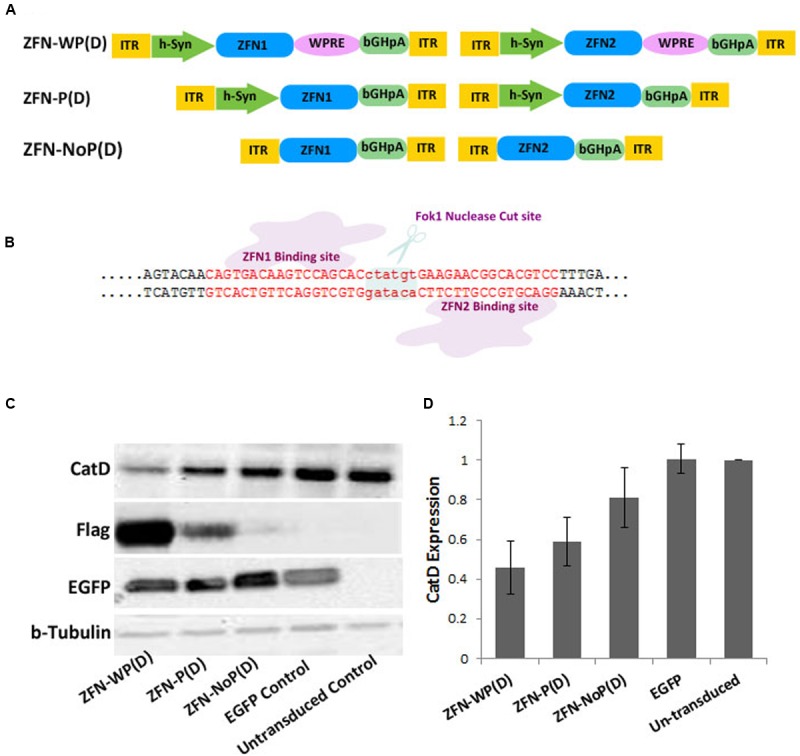
**Targeting of the CatD gene in cultured neurons by a 2-vector AAV delivery system. (A)** The + strand ZFN (ZFN1) and the – strand ZFN (ZFN2) are expressed from two separate vectors in different layouts: ZFN-WP(D), with hSyn promoter and WPRE; ZFN-P(D), with hSyn promoter but without WPRE; ZFN-NoP(D), with left ITR as promoter; ITR, inverted terminal repeat; WPRE, woodchuck hepatitis virus post-transcriptional control element; bGH-pA, bovine growth hormone polyadenylation site; D, double vector system. **(B)** Schematic illustrating of ZFN binding strategy to Cathepsin D locus. **(C)** Western blot analysis of AAV expressed flag-tagged ZFN using cell lysates from mouse cortical neurons at 21 day post-transduction. AAV-EGFP was used as transduction control. **(D)** Quantitative evaluation of band intensities of western blot analysis reveals more reduction of CatD protein levels by higher levels of ZFN expression. Data given as means from three independent experiments ±SEM.

**FIGURE 2:**
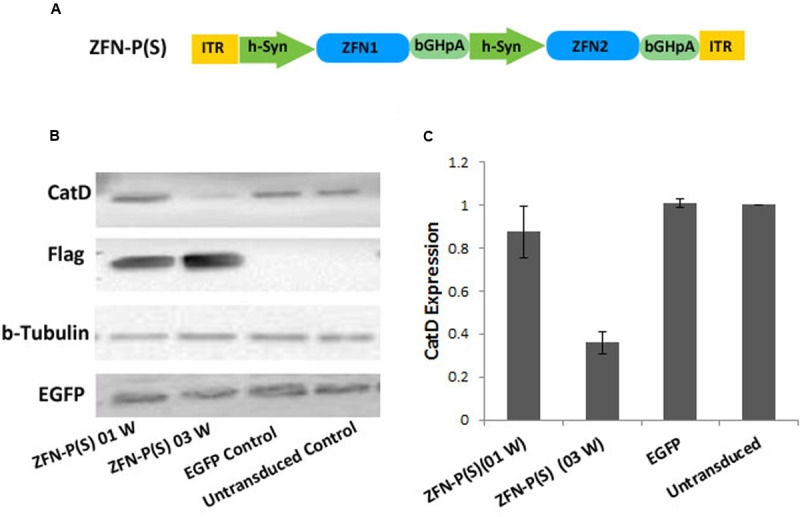
**Targeting of the CatD gene in cultured neurons by a 1-vector AAV delivery system. (A)** The + strand ZFN (ZFN1) and the – strand ZFN (ZFN2) are expressed from a single vector, each by its own hSyn promoter but without WPRE. ITR, inverted terminal repeat; WPRE, woodchuck hepatitis virus post-transcriptional control element; bGH-pA, bovine growth hormone polyadenylation site; S, single vector system; W, week. **(B)** Western blot analysis of AAV expressed flag-tagged ZFN using cell lysates from mouse cortical neurons at 7 or 21 days post-transduction. AAV-EGFP was used as transduction control. **(C)** Quantitative evaluation of band intensities of western blot analysis reveals that significant reduction of CatD levels was achieved at 21 days after transduction. Data given as means from three independent experiments ±SEM.

The pair of ZFNs targeting the mouse CatD gene was designed and functionally verified by Sigma Aldrich (Accession Numbers; CatD_ZFN1: KY862007 and CatD_ZFN2: KY862008). The target site of ZFN binding is shown in **Figure 1B**. Initially both ZFN constructs (ZFN1 = 3 zinc finger motifs targeting the plus-strand of *CatD* plus Fok1 nuclease domain and FLAG-tag, and ZFN2 = 3 zinc finger motifs targeting the minus-strand of *CatD* plus Fok1 nuclease domain and FLAG-tag) were cloned in separate AAV vector genomes in three different layouts: (1) under control of the human Synapsin 1 gene (hSyn) promoter and with WPRE as mRNA stabilizing element (Kügler, 2016), (2) under control of the hSyn promoter but without WPRE element, and (3) without hSyn promoter or WPRE. In the third construct, ZFN expression was driven by the basic promoter activity of the left AAV-2 inverted terminal repeat (ITR) (**Figure 1A**). For the 1-vector layout, both ZFNs were cloned head-to-tail under control of their own hSyn promoter, but without WPRE element due to size constraints (**Figure 2A**).

Recombinant AAV vectors of serotypes 6 (AAV6) and mosaic serotype 1/2 were produced and purified as previously described (Kügler et al., 2007).

### Neuronal Cell Culture, Western Blotting, and Ca^2+^ -Sensor Imaging

Primary cortical neuron / glia co-cultures were prepared from embryonic day 18 mouse pups as described (Kügler et al., 2001). Cultures were infected with AAV6 vectors on day 1 *in vitro* (DIV1) by diluting the proper amount of viruses in 10 μl of phosphate-buffered saline which was then directly added to cells grown in 24-well plates; 1 × 10^7^ vector genomes (vg)/250.000 cells of AAV6-ZFN1-WP(D) and AAV6-ZFN2-WP(D), AAV6-ZFN1-P(D) and AAV6-ZFN2-P(D), AAV6-ZFN1-NoP(D), and AAV6-ZFN2-NoP(D), along with AAV6-EGFP as transduction control were applied. To reduce CatD expression and secretion from glial cells, 1-β-D-arabinofuransylcytosine (ARA-C, 0.5 μM) was added to the cultures on DIV2 to transduction and medium was changed every 3rd day to minimize extracellular CatD. For western blots cells were harvested at day 22 post-transduction. For bicistronic expression of CatD-ZFNs, AAV6- ZFN1+2-P(S) was used at 1 × 10^7^ vg/250.000 cells and the cells were harvested after 7 or 22 days post-transduction. Blots were developed using the following primary antibodies: goat anti CatD mouse (R&D, AF1029); mouse anti Flag M2 (Sigma, F3165); mouse anti EGFP (Roche, 11814460001) and mouse anti β-tubulin (Sigma, T4026) followed by secondary anti-mouse and anti-rabbit HRP antibodies (Sigma-Aldrich). Tubulin served as loading control. Blots were imaged with ChemiDoc MP System with ImageLab 4.1 software (Bio-Rad), and quantified using ImageJ software 1.49.

Neuronal functionality was addressed through co-transduction of the eGFP based genetically encoded calcium indicator (GECI) GCaMP3.5 (Tian et al., 2009) and Ca^2+^ imaging was performed essentially as described (Mironov et al., 2009). Electrical stimulation was done using a SIU-102 (Warner Instruments) stimulus isolation unit and custom built function generator. Neurons were in all cases stimulated by emulating a train of 50 action potentials (AP; 10 Hz, 1 ms pulse-width, 100 mA current and 2 cm electrode separation) and data was evaluated using ImageJ 1.49 software.

### *In Vivo* AAV-ZFN Injection

All experimental animal procedures were conducted according to approved experimental animal licenses (33.9-42502-04-11/0408) issued by the responsible animal welfare authority (Niedersächsisches Landesamt für Verbraucherschutz und Lebensmittelsicherheit) and controlled by the local animal welfare committee of the University Medical Center Göttingen. Animals were housed in standard conditions in a dark/light cycle of 12 h, with free access to food and water. Two to three months old female CD-1 mice were anesthetized by intraperitoneal injection of ketamine (100 mg/kg) supplemented by xylazine (5 mg/kg) and positioned in a stereotaxic apparatus (Kopf Instruments, Tujunga, CA, USA). Viral vectors were stereotaxically injected in two deposits of 2 μl each containing in total 2 × 10^8^ vg [AAV1/2- ZFN1+2-P(S)] into the right striatum at the following coordinates relative to bregma: AP -0.01 mm, ML -2.1 mm, DV -3.7 mm and AP -0.09 mm, ML -1.5 mm and DV -3.7 mm. After the injection, the syringe was left in place for an additional 5 min to allow the diffusion of the viral vectors and minimize backflow.

### Immunohistochemistry and Microscopy

Mice were euthanized at 4, 8, and 16 weeks post-injection, and transcardially perfused with 0.1 M PBS, pH 7.4, followed by 4% (w/v) paraformaldehyde (PFA) in 0.1 M PBS. The brains were removed and post-fixed overnight in 4% (w/v) PFA followed by dehydration in 30% (w/v) sucrose prepared in PBS. Serial coronal sectioning was performed at a thickness of 30 μm using a cryostat (Leica Microsystems, Wetzlar, Germany). Free-floating sections were rinsed with Tris-buffered saline and subjected to immunohistochemistry as described (Tereshchenko et al., 2014). Antibodies used were as follows: rabbit anti-CatD (Abcam, ab75852); mouse anti Flag M2 (Sigma, F3165); rabbit anti-NeuN (Abcam, ab104225); rabbit anti-IbaI (Wakochem, 01919741), rabbit anti-GFAP (DAKO, Z0334). Cy2- or Cy3-conjugated secondary antibodies (Dianova, Germany) were used for visualization. Each group has seven mice and from each injected brain, 6–7 slices of injected striatum have been analyzed. Bright-field and fluorescent images were recorded on a Zeiss Axiovision microscope equipped with cooled CCD camera or with an ApoTome (Zeiss) and Axiovision 6 software. For confocal imaging, the images were captured using a laser-scanning confocal microscope (Leica Microsystems, Wetzlar, Germany) at 40 or ×100 magnifications and analyzed using the ImageJ software.

### Off-Target Predictions and Indel Detection Using Deep Sequencing

For the analysis of ZFN activity, genomic DNA was isolated from mouse neurons after 2 and 3 weeks of transduction with CatD- ZFNs and from control neurons either transduced with control ZFN (not designed against CatD) or the virus expressing ZFN1 or ZFN2 only (that alone are not capable of making DSB in DNA). ZFN activity was determined by deep sequencing at either on-target (mouse CatD) or off-target (1–4 off-target sites as predicted by Genbank homology search of CatD) sites for both the control and the ZFN treated neurons.

Deep sequencing was done at the Microarray and Deep Sequencing Core Facility (Transkriptomanalyselabor, TAL) of the University Medical Center Goettingen. Target regions were amplified from genomic DNA using specific PCR primers (Supplementary Table 1), amplicons were pooled in equimolar amounts and sequencing libraries were generated using TruSeq DNA PCR-free kit and protocol from Illumina. Libraries were pooled and sequenced on an Illumina Miseq using the Nano reagent kit and 2 × 150 bp paired-end configuration. Sequencing yielded between 37,600 and 141,400 combined reads per sample, the read quality was checked using the FastQC software (Babraham Bioinformatics^1^).

Sequencing reads were aligned to the genome sequence of Mus musculus (assembly version GRCm38) using BWA (Li and Durbin, 2009) in ‘mem’ mode and standard configuration. Duplicate reads were marked using Picard tools^2^. Indel frequencies were calculated by determining for each on-target/off-target region the number of reads with a particular number of insertions/deletions in the CIGAR string and comparing this to the total number of mapped reads per region. All reads were required to have a MAPQ value of at least 60 and duplicated reads were not counted.

### Statistical Analysis

All experiments were performed in at least three independent replicates. Data are expressed as means ± standard error of mean (SEM). Multiple comparisons were made by one-way analysis of variance test. The unpaired student’s *t*-test was used for comparison between two groups. Differences were considered significant at *P* < 0.05 and less.

## Results

### Neuronal *In Vitro* Expression of CatD-ZFNs by a 2-Vector System

In order to study ZFN-mediated gene ablation in neurons *in vitro*, we tested a pair of ZFNs against the murine CatD gene in cultured cortical neuron/glia co-cultures, isolated from Embryonic day 18 (E 18) mouse brains. Neurons in these cultures do not divide anymore, mature into fully differentiated cells and survive in culture for about 4 weeks. Thus, they allow for a relatively long-lasting expression of ZFNs within the target cells, which is important to study potential neurotoxic effects due to ZFN expression.

Zinc finger nucleases were expressed by means of AAV-6 vectors, which were added to the cell culture medium at div (day *in vitro*) 1, driving ZFN expression from the neuron-specific synapsin1 gene promoter (hSyn). As we were interested in investigating the effects of different amounts of ZFNs expressed in the neurons, we created three different expression systems. Each of them is based on a 2-vector approach, where the plus-strand ZFN is expressed from one AAV vector, and the minus-strand ZFN is expressed from a second AAV vector (**Figure 1A**). As shown by western blotting and detection of the Flag-epitope tag fused to each ZFN, the highest expression level was achieved with vectors expressing the ZFN from the hSyn under reinforcement by the woodchuck hepatitis virus post-transcriptional regulatory element [WPRE (Klein et al., 2002)]. About fivefold lower ZFN expression levels were achieved with vectors expressing the ZFN from a hSyn promoter in absence of the WPRE element, and again fivefold lower expression levels were achieved by omitting the hSyn promoter and exploiting the low-level promoter activity of the left AAV ITR (**Figure 1C**). In cell lysates collected at div 22 (i.e., 21 days after transduction with respective AAV vectors), CatD protein levels were reduced to 46% of controls by high level ZFN expression, to 59% of controls by medium level ZFN expression, and to only 81% of controls by low level ZFN expression (**Figure 1D**).

In these experiments a third AAV expressing EGFP from the hSyn promoter was used to label neurons for counting and morphological assessment. We found no evident reduction of neuron numbers or morphology by any level of ZFN expression (**Figure 4E**), and EGFP protein expression levels were not affected by concomitant co-expression of ZFNs (**Figure 1B**).

These results revealed that the level of CatD-ZFN expression is directly related to the reduction of CatD protein expression, indicating that higher levels of ZFNs provide more efficient induction of DNA cleavage. However, despite use of Ara-C to limit growth of glial cells and medium change every 3 days to reduce the amount of secreted CatD available to become re-uptaken by neurons in which a gene deletion was already produced, a complete depletion of CatD protein levels could not be achieved over the time course of the experiment. This may be due either to CatD protein supply from glia to neurons, the extraordinary long half-life of CatD (Faust et al., 1987) or insufficient diploid gene deletion, probably due to limited co-transduction efficacy of the 2-vector system.

### Neuronal *In Vitro* Expression of CatD-ZFNs by a 1-Vector System

While AAVs are favorable gene transfer systems, they are hampered by limitations in transgene capacity, which, including transcriptional control elements, should not exceed about 4700 bp. Thus, the above-described 2-vector system with highest efficacy (i.e., including the WPRE element within the expression cassettes) cannot be fit into a single AAV vector genome. None the less, we argued that probably the more even transduction achieved with a 1-vector system might positively impact on ablation of the CatD gene. To address this question we generated a single vector genome for expression of both ZFNs from individual hSyn promoters, but in absence of WPRE element (**Figure 2A**). This vector was tested under the same conditions as the 2-vector systems, and demonstrated a significantly (*p* < 0.001) stronger degree of CatD reduction (**Figures 2B,C**) at 21 days after transduction. As compared to the corresponding 2-vector system, the optimized 1-vector system was about two-fold more efficient, as it achieved a reduction in CatD protein levels to 35% of controls as compared to 59% of controls achieved with the respective 2-vector system. Furthermore, this experiment demonstrated that at 1 week after transduction no significant reduction of CatD protein levels were achieved, again suggesting that the protein’s stability and compartmentalization results in delayed depletion kinetics.

### Indel Frequency of CatD Locus after ZFN Administration in Mouse Neurons

Commercial design of ZFN pairs targeting a specific gene locus is based more on specificity than on efficacy, and accordingly the mouse DNA sequence the most closely related to the target sequence of the *CatD* gene showed already 18 mismatches. No currently available data suggest that such sites may serve as off-target sites (Cradick et al., 2011; Lee et al., 2016). To further confirm the off-target cleavage incredibility, we selected four off-target sites predicted by homology search tool for deep sequencing transcriptome analysis.

**FIGURE 3:**
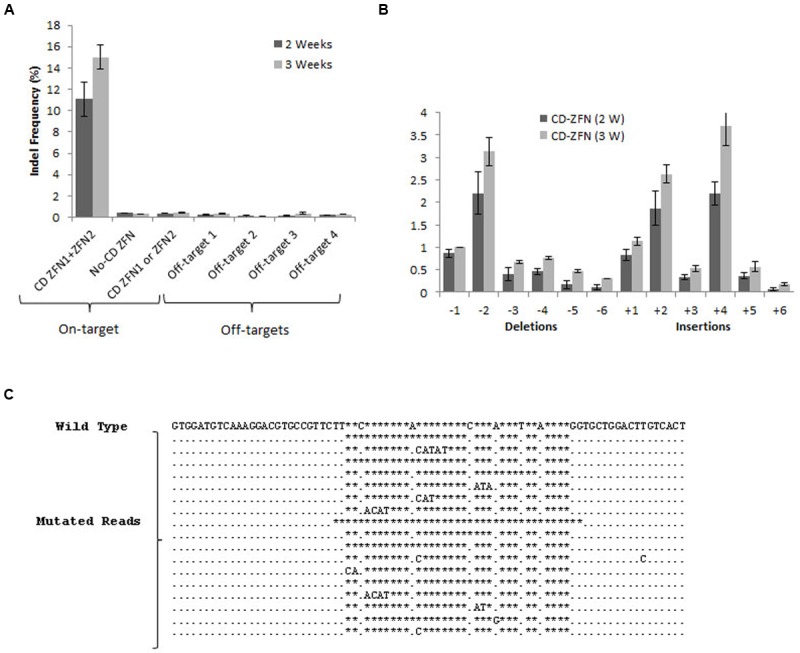
**Frequency and pattern of gene modifications following ZFN administration in mouse neurons. (A)** On-target and off-target indel frequencies for the CatD gene after 2 and 3 weeks of ZFN expression. Amplicons for the target sequence of CatD were obtained from the DNA isolated from the neurons transduced with ZFN against CatD. Neurons transduced with the ZFN1 and ZFN2 individually and those with non-CatD ZFN served as negative control. Off-target amplicons were obtained from the DNA collected from the neurons expressing CatD ZFN. **(B)** Distribution of indel length (Number of deletions and insertions) in CatD gene after 2 and 3 weeks of ZFN expression. For **(A,B)**, error bars reflect SEM from three biological replicates performed on independent experiments. **(C)** Representative examples of genomic DNA sequences at CatD locus that are modified following ZFN transduction. The unmodified genomic site is the first sequence, followed by the most abundant sequences containing deletions and insertions. The stars represent gaps, a dot for a match on the same strand.

To estimate the indel frequency, both on-target CatD as well as predicted off-target sites (Supplementary Table 1) were amplified from genomic DNA isolated from mouse cortical neurons treated either by CatD-ZFN or by control ZFN. The indel frequencies were assayed by high-throughput DNA sequencing. After 2 weeks, the frequency of on-target DNA modification is about 11% that is increased to 15% after 3 weeks (**Figure 3A**). Indel patterns indicated mostly 1–2 base deletions and 2–4 base insertion within the on-target CatD locus. More than five bases deletion or insertions were almost negligible (**Figures 3B,C**). The quantification of off-target indel frequencies revealed an extremely low level of off-target modification without any difference between 2 and 3 weeks (**Figure 3A**).

### Neuronal ZFN Expression Does Not Impact on Essential Neuronal Functionality

Although our ZFN did not exhibit significant off-target cleavage in the four sites chosen for analysis, this does not guarantee that there is not non-specific cleavage at other off-target sites in the mouse genome. ZFN targeting is known to involve context dependency for sequence binding, and off-target cleavage may occur more efficiently at sites with less sequence homology than the closest matches (Gabriel et al., 2011). We therefore decided to address this issue by investigating an essential function of neurons, namely their ability to respond to electrical stimulation.

**FIGURE 4:**
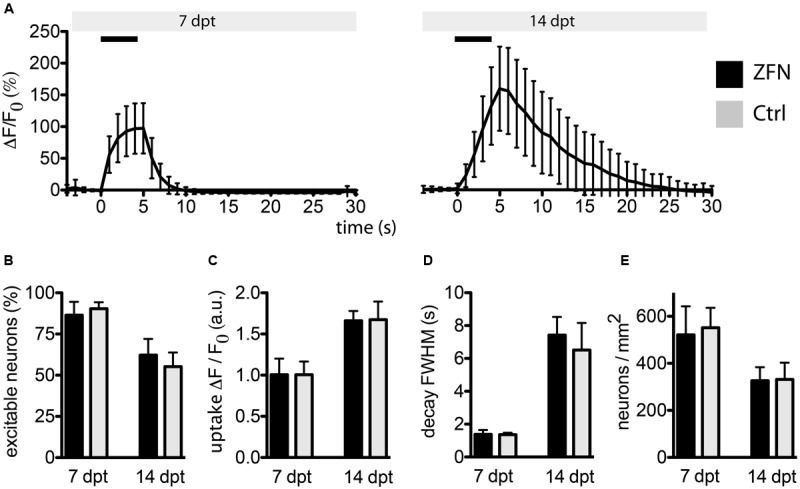
**Zinc finger nuclease expression in primary neurons does not affect essential neuronal functionality.** Neurons co-transduced with 1-vector ZFN construct and GCaMP3.5 were subjected to field stimulation (FS). **(A)** Typical traces obtained from neurons at 7 or 14 days post-transduction are shown, with black horizontal bars representing the 5 s stimulus at 10 Hz. **(B)** The fractions of excitable neurons were not different between ZFN-expressing neurons or controls. **(C)** The maximum uptake of calcium upon FS was not different between ZFN expressing neurons and controls. **(D)** Calcium decay times after FS were not different between ZFN expressing neurons and controls. **(E)** The number of neurons per mm^2^ were not different between ZFN expressing neurons and controls.

We co-expressed the genetically encoded Ca^2+^ sensor GCaMP3.5 together with the 1-vector CatD-ZFN construct or with an empty control vector, allowing to record neuronal excitability and ion handling capabilities by life cell imaging. We quantified the fraction of neurons responsive to a short electrical stimulation (field stimulation for 5 s at 10 Hz with 100 mA), the kinetics of cytosolic Ca^2+^ influx as a response to APs (i.e., opening of voltage-gated Ca^2+^ channels following membrane depolarization) and the kinetics of decay of cytosolic Ca^2+^ as a measure of neuronal fitness to handle essential ion fluxes. All these parameters were identical between neurons expressing the CatD-ZFNs and control neurons at either 7 or 14 days post-transduction (**Figures 4A–D**), indicating that no evident impairment of essential neuronal functionality was caused by robust ZFN expression.

It should be noted that due to the facts that (i) expression of CatD-ZFNs resulted in robust but not complete elimination of CatD protein in neurons, and (ii) already minute amounts of residual CatD in neurons are sufficient to maintain lysosomal functions, these neurons were not affected by pathophysiology caused by targeting the *CatD* gene.

### *In Vivo* Application of CatD-ZFNs in Mouse CNS Neurons

**FIGURE 5:**
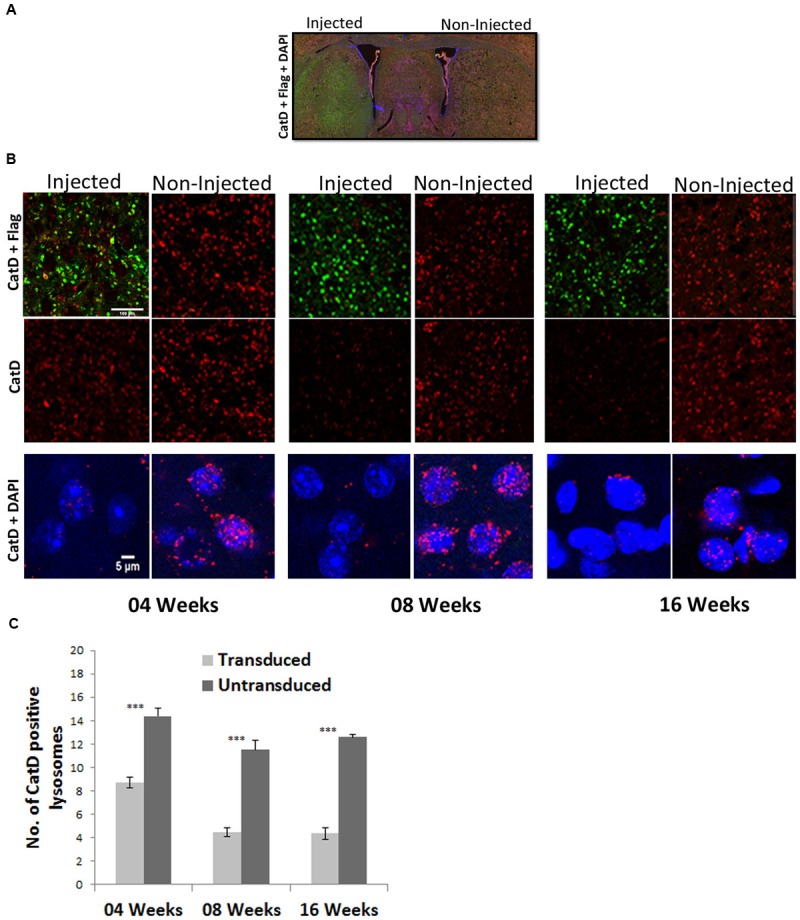
**Targeting of the CatD gene in striatal neurons of the adult mouse brain by a 1-vector AAV delivery system. (A)** Overview of coronal sections showing flag-tagged ZFN (green) expression in the striatum. **(B)** Striatal tissue sections were prepared at 4, 8, and 16 weeks after the injection of single AAV1/2-ZFN vector and were immunohistochemically stained for CatD (red), the Flag-tag fused to each ZFN (Green; upper two rows of panels) and CatD (red) and DAPI (blue) in higher magnifications (lower row of panels). Images were recorded on a confocal microscope. Scale Bar = 100 μm for the upper two panels and 5 μm for the lower row of panels. **(C)** Quantification of Cat-D positive lysosomes in transduced and untransduced sides of the striatal area. ^∗∗∗^Indicates *p* < 0.0001 Student’s *t*-test; error bars = SEM.

Next we investigated expression of CatD-ZFNs *in vivo*, in striatal neurons of the mouse brain. For this experiment we used a hybrid AAV-1/2 vector for expression of both ZFNs in the 1-vector layout (**Figure 2A**), rather than the AAV-6 vector exploited for *in vitro* studies. AAV-1/2 vectors allow for wider spread of viral particles through brain tissue as compared to AAV-6 (Wu et al., 2006; **Figure 5A**). Primary goal of this experiment was induction of a CatD-KO phenotype in transduced neurons, i.e., the generation of auto-fluorescent ceroid accumulations (Koike et al., 2000).

However, it should be noted that the neuron-specific hSyn promoter does not allow for CatD-ZFN expression in glial cells, and that even AAV-1/2 vectors transduce only a limited brain volume upon intracranial injection. Thus, it is likely that glial cells in the transduced area and neurons remote from AAV transduction can supply even those neurons that receive a diploid *CatD* gene deletion with a certain amount of CatD through extracellular secretion (Shevtsova et al., 2010). It was therefore not too surprising that no ceroid accumulation could be detected in ZFN expressing neurons.

None the less, in neurons immuno-reactive for the flag-tag fused to ZFNs, a robust downregulation of CatD immuno-reactivity was detected (**Figure 5B**). As the majority of CstD immuno-reactivity was found in lysosomes, we quantified CatD-immunoreactive lysosomes and found that at 4 weeks after AAV-CatD-ZFN transduction their number was reduced to 60% of controls, while at 8 and 16 weeks CatD lysosomal immuno-reactivity was reduced to about 35–40% of controls (**Figure 5C**). As controls we used neurons from the corresponding area of the contralateral hemisphere. The staining intensity of lysosomal CatD was reduced by almost 80–90% as compared to control neurons not expressing the ZFNs, but as immunohistochemistry is at best a semi-quantitative technique we used only the number of immunoreactive lysosomes as a quantitative measure. These results showed that the maximum downregulation of CatD protein levels took up to 8 weeks, but that further expression of CatD-ZFNs for another 8 weeks did not significantly improve this effect.

**FIGURE 6:**
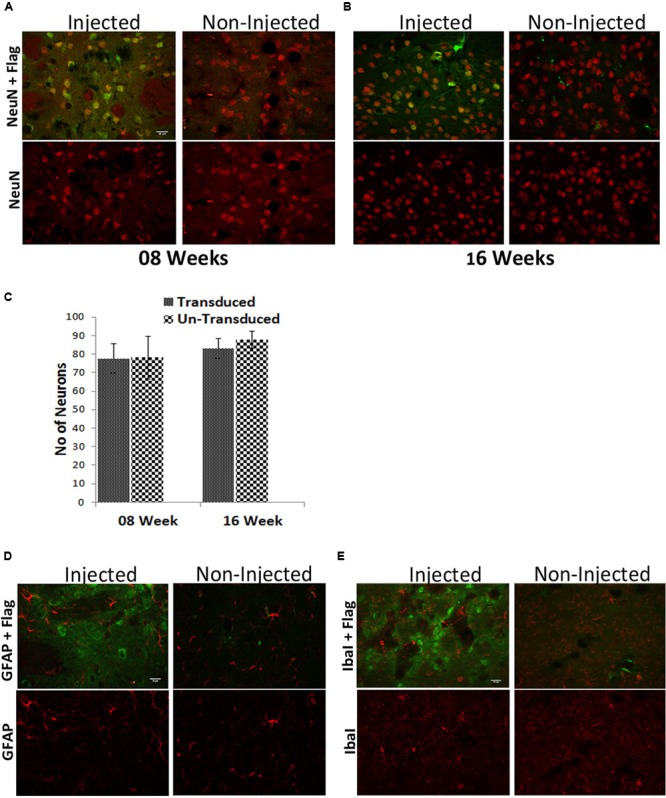
**Long-term ZFN expression in the adult mouse brain is safe. (A)** Mouse striatal neurons were stained for the neuronal marker NeuN (red) and the ZFN-fused Flag-tag (green) after 8 weeks **(A)** or 16 weeks **(B)** of CatD-ZFN expression from the one-vector AAV. Stereological counting revealed that at both times the number of neurons is not different in AAV-CatD-ZFN transduced hemisphere versus the contralateral control hemisphere **(C)**. Brain sections were also stained for the astroglial antigen glial fibrillary acidic protein (GFAP, red) as shown in **(D)** and for the microglial antigen Iba1 (red) in **(E)**. No evidence for astroglial or microglial activation was detectable in AAV-CatD-ZFN transduced hemisphere as compared to the contralateral control hemisphere. Scale bar = 20 μm.

In terms of safety of ZFN expression, we analyzed if long-term presence of the genetic scissors would result in any neuron loss, and found that at 8 (**Figure 6A**) and 16 (**Figure 6B**) weeks after AAV-CatD-ZFN transduction numbers of NeuN positive neurons were identical in the transduced hemisphere and the contralateral, control side (**Figure 6C**). Furthermore, we found no signs of microglial or astroglial activation other than that caused by the mechanical lesion of vector injection (**Figures 6D,E**), suggesting that long-term expression of ZFNs including their bacterial nuclease moiety by means of AAV vectors is well tolerated by mouse CNS neurons *in vivo*.

## Discussion

The main technical issue addressed in the present study is that of tolerance against long-term expression of a functional pair of ZFN gene scissors in rodent CNS neurons. Our results demonstrate that in cultured neurons *in vitro* robust long-term ZFN expression did not impact on essential neuronal functionality. Likewise, after 4 months of CatD-ZFN expression in mouse CNS neurons *in vivo*, no signs of neurotoxicity were evident. We did not detect any neuron loss, inflammation or astrogliosis due to AAV-mediated ZFN expression, thereby demonstrating that a pair of ZFNs can serve as safe gene deletion tools in the CNS. This is the first report on functional ZFN expression in the brain by AAV vectors. A monomeric zinc finger domain coupled to a kox1 repressor domain have been delivered to mouse brain by means of AAV vector earlier, however, this molecule lacks a nuclease moiety and acts by steric hindrance of transcription rather than by inducing genetic deletions (Garriga-Canut et al., 2012). Our study is also the first report on any long-term expression of a gene editing tool in the CNS. A recent report on AAV-mediated Crispr/Cas gene targeting in the mouse brain showed successful reduction of the target proteins to a level comparable to that achieved in our study, but was conducted for only 2 weeks, thus lacking long-term analysis of potential neurotoxic effects (Swiech et al., 2015).

It was the main biological aim of the present study to achieve a localized, neuron-specific KO of the CatD gene in mouse brain neurons, in order to study potential neurotoxic effects of the ceroid accumulation caused by loss of CatD activity. As CatD -/- mice die at p21 (Saftig et al., 1995), this ZFN-mediated approach offered a unique chance to investigate neuropathology caused by CatD ablation in the adult CNS. Clearly, this goal was not met, as expression of CatD-ZFNs over prolonged periods of time were not sufficient to eliminate CatD completely from transduced neurons. However, since a complete depletion of CatD from neuronal lysosomes would have induced neuropathology, a valid long-term assessment of consequences caused by ZFN expression itself would not have been possible.

Pulse-chase studies in Xenopus oocytes have shown that CatD has an extraordinarily long half-life (Faust et al., 1987), which may explain the fact that in cultured neurons CatD protein levels could not be completely depleted. Low-level residual CatD immunoreactivity in lysosomes of mouse brain striatal neurons may be due to two reasons: (1), glial cells in the transduced area, or neurons remote from the transduced area, continuously secrete a certain amount of their synthesized CatD into the extracellular fluid. Mannose-6-phosphate receptors on ZFN expressing neurons will thus be able to capture this CatD and direct it into lysosomes irrespective of a successful gene knock-out. It is long known that lysosomal hydrolases are secreted to a certain extent and can be endocytosed by remote cells. This mechanism is used in pre-clinical therapy models for, e.g., ß-glucuronidase deficiency, where diffused enzyme is detected within brain cells far remote from application of the viral expression vector (Liu et al., 2005). The recent finding that CNS expressed CatD even reaches peripheral lymph nodes (Shevtsova et al., 2010) argues for this “spread hypothesis.” CatD is heavily secreted from certain cancer cells, but also from many normal tissues (Piwnica et al., 2006) including neurons. CatD secreted from CNS neurons is capable of diffusing over large distances and is re-uptaken by remote neurons as well as by peripheral tissues (Shevtsova et al., 2010).

Alternatively, it may be that the ZFN-mediated gene ablation is not diploid in all transduced neurons, allowing a certain proportion of these transduced cells to produce CatD from the non-affected allele and to distribute the protein through secretion and uptake through mannose-6-phospate receptors to the other ZFN-expressing neurons. The uniformly low-level staining for CatD in ZFN-positive neurons argues against this hypothesis. However, recent results obtained from CRISP/Cas transduced neurons in the mouse brain suggest, that the diploid gene disruption efficacy with this type of genetic scissor can be highly variable, depending on the locus targets. Diploid indels were obtained in between 18 and 68% of neurons targeted for their *Mecp2* or *Dnmt1* loci (Swiech et al., 2015). While such a gene knock-down efficacy may be sufficient to induce phenotypes under certain conditions, it is generally not more efficient than RNAi-mediated gene silencing, and thus not equivalent to a classical gene knock-out obtained by genetic manipulation of oocytes.

Our finding of significantly better CatD depletion in case of expressing both ZFNs from a single vector, rather than trying to enhance expression levels with a 2-vector system, suggest that such single vector layout should be used for future studies aiming at achieving the maximal indel frequency. The recent introduction of a smaller Cas9 molecule from *S. aureus* (Ran et al., 2015) will also enable this strategy for Crispr/Cas expressing vectors.

Growing evidence suggests that ZFN and CRISPR/Cas mediated gene targeting substantially increases the frequency of successful gene replacement strategies. Such studies have so far mainly been conducted by AAV mediated delivery of ZFNs or CRISPR/Cas into hepatocytes. The relatively low insertion/deletion (indel) frequency of transduced cells obtained under these conditions was sufficient for therapeutically relevant gene replacement events (Landau et al., 2016). If aiming for cutting down genes to produce localized, cell-type specific KO phenotypes, a high diploid indel frequency is absolutely necessary. However, indel frequency from the *in vivo* applications of CRISPR/Cas and ZFN is highly variable depending on the candidate sequence, specific location and delivery procedures. In the present study, we observed 10–15% indel frequency after two and 3 weeks of CatD-ZFN administration to the neurons, respectively. This increase in the indel frequency from 2 to 3 weeks indicates the more efficient knock down effect of ZFN with time. This is comparable to the indel frequency of earlier results obtained by SMRT sequencing and the Surveyor assay of ZFN application against albumin locus in liver (12–17%, Sharma et al., 2015) and HBV gene (16–40%) (Weber et al., 2014). CRISPR/Cas also showed a similar range of indel frequency, e.g., 5% indel frequency against apolipoprotein (*Apob*) gene was reported in liver after 1 week but 40% against *PCSK9* locus after 2–4 weeks (Ran et al., 2015). Likewise, 4–7% indels were observed at *Pten* and *p53* loci in the liver on CRSPR/cas delivery (Xue et al., 2014).

## Conclusion

This study is the first attempt to express a pair of functional ZFNs from a single AAV vector in the rodent CNS. We could demonstrate that long-term CatD-ZFN expression in striatal neurons did not cause detectable neuro-degeneration, inflammation or astrogliosis, suggesting safety of this approach in the mouse CNS. Although depletion of the target protein was robust, a complete physiological KO could not be achieved. This is in agreement with other recently published genetic scissors like AAV-expressed Crispr/Cas in rodent CNS, suggesting that the efficacy of these tools to produce diploid gene knock-outs still needs improvements. Since such studies are only now emerging, it is still too early to compare efficacy for diploid indel formation in brain neurons between Crispr/Cas and ZFNs. Future studies targeting the same gene locus with either one tool in a side-by-side approach may clarify this issue.

## Author Contributions

MZ: Experimental design and work acquisition, Data analysis and interpretation, drafting the work and finalizing the manuscript. JT: Ca^+^ sensor imaging, Help in ImageJ analysis. MB: Critical review of the work and final approval to publish. SK: Project design, Data interpretation and intellectual discussions, drafting and critical revision of the article.

## Conflict of Interest Statement

The authors declare that the research was conducted in the absence of any commercial or financial relationships that could be construed as a potential conflict of interest.

## Footnotes

1 www.bioinformatics.babraham.ac.uk/projects/fastqc/

2 broadinstitute.github.io/picard/
