# Supplementary material for: Long-Term Assessment of AAV-Mediated Zinc Finger Nuclease Expression in the Mouse Brain
Source: Front Mol Neurosci. 2017 May 23;10:142. doi: 10.3389/fnmol.2017.00142 (PMC5440507; doi:10.3389/fnmol.2017.00142)
Supplement: Supplementary file 1 [file Table_1.PDF]

Supplementary Table1

On-target and off-targets for *CatD* identified by homology prediction

Gene	Forward primers	Reverse primers	Chromosome	Indel Freq % (2 weeks)	Indel Freq % (3weeks)
Cathepsin D (On target)	CTACTGGCCCCACAGCAC	CCCCTCAGCTGTAGTTGCTC	Chr 7	11.0686	15.00498
	AAATTGGAGAGTTAGCCTGAGC	CTCTGAGCTCCAAGGACAGG			
Rab 17 (Off-target1)	CCTCTCAGGGAGCTGCTAAG	GGTGGGAAGTGACCCTACCT	chr 1	0.200006	0.351087
	GGTCAGGATGACGCACTAGG	CGAGTGATGTCATAAACCAGGA			
clarin-1 isoform 3 (Off-target2)	GGCTCCCCTGACTGAATTCT	TCCAGGAAGCTCACCATTTT	chr 3	0.095061	0.092812
	AAGCCAAAGGCTGTGTGTTTA	CTGGGATCCACCTGACTCTG			
lysophosphatidic acid receptor (Off-target3)	CACAAGGAAGACAAGAACAAAGAA	CACACACACACACACAAATCAA	chr X	0.141835	0.347684
	AACAAGATGACAATAGTGAGATCCA	CACAAATCAATAAATGTGGTCTACC			
cell adhesion molecule 2 isoform 2 precursor (Off-target4)	AACTTTGAACCTCGGCACA	AAAGGCATTTTGTGCCATTTA	chr 16	0.226452	0.256297
	AAATTGGAGAGTTAGCCTGAGC	TTCAGTCAAATTAAAGGCTGGA			
